# Broad Virus Detection and Variant Discovery in Fecal Samples of Hematopoietic Transplant Recipients Using Targeted Sequence Capture Metagenomics

**DOI:** 10.3389/fmicb.2020.560179

**Published:** 2020-11-17

**Authors:** Suze A. Jansen, Wouter Nijhuis, Helen L. Leavis, Annelies Riezebos-Brilman, Caroline A. Lindemans, Rob Schuurman

**Affiliations:** ^1^Division of Pediatrics, University Medical Center Utrecht, Utrecht, Netherlands; ^2^Department of Stem Cell Transplantation, Princess Máxima Center for Pediatric Oncology, Utrecht, Netherlands; ^3^Department of Medical Microbiology, University Medical Center Utrecht, Utrecht, Netherlands; ^4^Department of Rheumatology and Clinical Immunology, University Medical Center Utrecht, Utrecht, Netherlands

**Keywords:** targeted sequence capture, next generation sequencing, virus, clinical samples, graft-versus-host disease, molecular diagnostics, HSCT

## Abstract

Pediatric allogeneic hematopoietic stem cell transplantation (HSCT) patients often suffer from gastro-intestinal (GI) disease caused by viruses, Graft-versus-Host Disease (GVHD) or a combination of the two. Currently, the GI eukaryotic virome of HSCT recipients remains relatively understudied, which complicates the understanding of its role in GVHD pathogenicity. As decisions regarding immunosuppressive therapy in the treatment of virus infection or GVHD, respectively, can be completely contradicting, it is crucial to better understand the prevalence and relevance of viruses in the GI tract in the HSCT setting. A real time PCR panel for a set of specific viruses widely used to diagnose the most common causes of GI viral gastroenteritis is possibly insufficient to grasp the full extent of viruses present. Therefore, we applied the targeted sequence capture method ViroCap to residual fecal samples of 11 pediatric allogeneic HSCT recipients with GI symptoms and a suspicion of GVHD, to enrich for nucleic acids of viruses that are known to infect vertebrate hosts. After enrichment, NGS was applied to broadly detect viral sequences. Using ViroCap, we were able to detect viruses such as norovirus and adenovirus (ADV), that had been previously detected using clinical diagnostic PCR on the same sample. In addition, multiple, some of which clinically relevant viruses were detected, including ADV, human rhinovirus (HRV) and BK polyomavirus (BKV). Interestingly, in samples in which specific PCR testing for regular viral GI pathogens did not result in a diagnosis, the ViroCap pipeline led to the detection of viral sequences of human herpesvirus (HHV)-7, BKV, HRV, KI polyomavirus and astrovirus. The latter was an only recently described variant and showed extensive sequence mismatches with the applied real time PCR primers and would therefore not have been detected if tested. Our results indicate that target enrichment of viral nucleic acids through ViroCap leads to sensitive and broad possibly clinically relevant virus detection, including the detection of newer variants in clinical HSCT recipient samples. As such, ViroCap could be a useful detection tool clinically, but also in studying the associations between viral presence and GVHD.

## Introduction

Immunodeficient patients, and in particular allogeneic stem cell transplant (HSCT) recipients, experience a high incidence of gastro-intestinal (GI) symptoms such as nausea and diarrhea. There are multiple causes that can underlie these complaints. Firstly, post-transplant patients are severely lymphopenic and therefore prone to a more severe course of viral infections, many of which circulate among healthy children (Pochon and Voigt, 2018). Secondly, HSCT recipients are at risk of developing acute intestinal Graft-Versus-Host Disease (GVHD). This is an ultra-complex, life-threatening condition that can only be treated with additional immunosuppressive therapy. Despite matching for HLA, donor immune cells co-transplanted with the graft recognize the patient’s tissues as foreign and launch an inflammatory response causing damage to multiple organs. Severe GI-GVHD (grade 3–4) (Glucksberg et al., 1974) is associated with a high mortality risk, due to organ damage directly (wasting, malnutrition), or secondary to GVHD-therapy-related induced suppression of immune cells. The combination of enteric viral presence, a fragile and suppressed immune system and GI damage by recent chemotherapy and/or GVHD, provides a challenging treatment task for the clinician. Especially since decisions regarding immunosuppressive therapy in the treatment of virus infections or GVHD can be highly divergent. To complicate matters further, intestinal viral presence, even asymptomatic, has been shown to predispose for intestinal GVHD and compromise patients’ outcome (van Montfrans et al., 2015). Given the above, the identification and characterization of viruses is important for dedicated treatment in HSCT recipients with both GI symptoms and a clinical suspicion of GVHD. Regular monitoring can be used to tailor immunosuppressive therapy or warrant antiviral treatment (Feghoul et al., 2015). In addition, it could provide further insight into the association of viral presence and the development of GVHD.

Thus far, real-time polymerase chain reaction (real-time PCR) has been the gold standard for clinical diagnosis of viral infections (Edwards and Gibbs, 1994). Despite its unprecedented sensitivity, speed and cost-effectiveness, the technology is restricted to only detecting the specific primer-directed virus and limited in identifying and further characterizing virus variants which are genetically divergent from the original species. The unbiased approach of next generation sequencing (NGS) technology (reviewed in Shendure and Ji, 2008; Barzon et al., 2011) overcomes these limitations, albeit at the cost of speed and, more importantly, some detection sensitivity. The sequences reported by NGS in clinical samples are often dominated by those of human origin, which hinders the ability to detect viral nucleic acids in particular when present at low abundance (Daly et al., 2011).

Several methods have been described to improve NGS sensitivity for the detection of virus in clinical samples. These methods include low speed centrifugation and filtration to remove cellular debris, ultra-centrifugation to collect virus particles, nuclease treatment to deplete unprotected (human) DNA and/or RNA and viral expansion in culture. As an alternative to DNA depletions, enrichment strategies have been proposed in which the viral nucleic acids are specifically captured by hybridization with probe libraries. Capture techniques have gained popularity over the past decades in human genome diagnostics to search for rare mutations and disease causing variants (Hodges et al., 2007; Choi et al., 2009; Mamanova et al., 2010). More recently, targeted enrichment strategies were successfully implemented for the identification of virus in human samples (Brown et al., 2016; Gaudin and Desnues, 2018; O’Flaherty et al., 2018; Cummings et al., 2019; Metsky et al., 2019; Paskey et al., 2019), including the ViroCap approach described by Wiley and colleagues (Wylie et al., 2015). This NGS hybridization-based capture technique consists of a large panel of probes spread across the genomes of 34 families of DNA and RNA viruses, including 337 species, that infect vertebrate hosts (Wylie et al., 2015). The probes were designed such that various regions of a species genome are covered and can therefore enrich for known viruses as well as for genetically similar new variants.

We applied ViroCap technology to broadly detect virus in stored stool samples of 11 clinical pediatric HSCT recipients with GI-symptoms that were suspected of GVHD.

## Materials and Methods

### Collection and Storage of Clinical Samples

According to standard clinical protocol, stool samples from clinical patients with gastro-intestinal symptoms suspected of GI-GVHD after HSCT were collected in containers without additives for diagnostic viral PCR testing. Patients had received a related sibling bone marrow (BM) graft, a 10/10 HLA-matched BM, or an unrelated cord-blood (CB) transplantation. Residual fecal material was stored at –80°C within 1 h after collection and retrospectively included for analysis with ViroCap under a protocol approved by the University Medical Center Utrecht Medical Research Ethics Committee. Informed consent was obtained for the use of clinical data of included HSCT recipients.

### Nucleic Acid Extraction and Reverse Transcription

Approximately 100 mg of fecal material was added to 1 ml of Stool Transport and Recovery (STAR) buffer (Roche Diagnostics), vortexed and subsequently centrifuged at 17,000 g for 1 min. 500 μl of the supernatant was used for total RNA and DNA extraction with the MagnaPure 96 (Roche Diagnostics) automated nucleic acid isolation system and MagnaPure 96 DNA and Viral NA Large Volume Kit (Roche Diagnostics) according to the Viral NA Universal 4.0 Protocol. The purified nucleic acid elution volume was set to 50 μl.

cDNA synthesis with TaqMan^TM^ Reverse Transcription Reagents supplemented with random hexamers (Applied Biosystems, Foster City, CA, United States) was performed essentially according to manufacturer instructions with the following incubation steps: 10 min at 25°C, 30 min at 48°C, 5 min at 95°C and subsequent hold at 4°C. 20 μl of eluate was used per cDNA reaction. After cDNA synthesis, the sample was pooled with the original sample eluate for further processing. (c)DNA concentrations were measured using the Qubit 2.0 and the Qubit DS DNA HS Assay.

### Enzymatic DNA Fragmentation and Library Preparation

Fragmentation of the DNA in the extraction eluates was achieved enzymatically using the KAPA Hyper Prep Kit (Roche) and a 20 min incubation time at room temperature (RT). Subsequently, library preparation was performed using the KAPA Dual-Indexed Adapters Kit and the SeqCap EZ HyperCap Workflow (Nimblegen). Adapter ligation was followed by two sequential bead clean up steps, using the AMPure XP reagent (Beckman Coulter, Indianapolis, IND, United States). Unique adapter barcodes were used to be able to identify the DNA sequences for each clinical sample. The (c)DNA libraries of up to a maximum of 10 samples plus a negative PBS control were pooled at equal concentrations. The 11 clinical samples described here were processed in 2 separate runs.

### Sequence Enrichment Using the ViroCap Probe Library

Viral sequence enrichment was achieved using the ViroCap massive sequence enrichment procedure and probe design described earlier (Wylie et al., 2015). In brief, to block non-specific hybridization, 5 μl Cot DNA and 2 μl Hypercap Universal Blocking Oligos (Roche Diagnostics, Plaesanton, CA, United States) where added to the pooled sample libraries. After an Ampure bead cleanup the sample pools were eluted in 10.5 μl Hybridization Buffer (Roche Diagnostics, Plaesanton, CA, United States) and a single unit (4.5 μl) of biotinylated ViroCap probes was added (288 ng in the 1st run, 383 ng in the 2nd run) for hybridization. The hybridization reactions were incubated at 47°C in a thermocycler with a heated lid set to 57°C to prevent evaporation for a minimum of 48 hrs. Subsequently, the hybridized DNA was bound to previously washed Streptavidin-magnetic capture beads at 47°C for 15 min. Following magnetic capture and multiple washing steps the DNA samples were amplified by LM-PCR and eluted from the capture beads using AMPure XP beads. The sample library pools were then treated with 0.2 N NaOH according to the MiSeq System Denature and Dilute Libraries Guide protocol (Illumina, San Diego, United States). Phix DNA (Illumina, San Diego, United States) was added to each sample pool at a final concentration of 1%. Sequencing was performed on a MiSeq system (Illumina, CA, United States), using the MiSeq reagent kit V3 for 2 × 300 cycles.

### Metagenomic Sequence Data Analysis and Result Confirmation

The FASTQ files generated by the MiSeq system were analyzed using the Genome Detective Viral Metagenomics Data Analysis Pipeline, version 1.111^1^. Viral sequences identified and reported by Genome Detective were subsequently checked and confirmed by direct alignment of the FASTQ file with a reference sequence of the respective virus, using Geneious sequence analysis software, version 9.1.6^2^. In addition, where possible, confirmation of the presence of the pathogen was performed by real time PCR.

## Results

### Samples of Clinical GVHD Patients

Stored, residual stool samples of 11 pediatric patients, five females and six males, that had undergone an allogeneic HSCT for a variety of malignant and non-malignant diseases were used for ViroCap analysis (Table 1). All patients suffered from GI-symptoms suspected of gut GVHD, enteric virus infection or a combination of both. Ten patients were diagnosed with GI-GVHD ranging from grade 1 to 4 according to consensus guidelines (Glucksberg et al., 1974), whereas the histological findings of the gut biopsy of 1/11 patients (patient 3) did not meet the requirements for a gut GVHD diagnosis. Nonetheless, patient 3 remained suspected of gut GVHD based on skin GVHD in combination with GI-symptoms.

**TABLE 1:** Patient demographics.

**Patient no.**	**Gender**	**Age at SCT (years)**	**SCT indication**	**Graft**	**Conditioning**	**Virus feces prior to GVHD**	**GVHD timing (days)**	**GVHD gut grade**^ b^	**GVHD overall grade**^*b*^	**GVHD therapy**	**EFS**	**Survival**
1	M	3	T-ALL	5/6 CB	ATG, Bu-Flu	Yes	28	1	2	Pred, CsA	Relapse	Deceased
2	F	13	PID	6/6 CB	RTX, ATG, Bu-Flu	Yes	37	3	3	Pred, CsA, tacrolimus, MMF, sirolimus, MSC 3x, monoclonals	TRM: MOF	Deceased
3	M	1	PID	5/6 CB	ATG, Bu-Flu	Yes	20	0	2	Pred, CsA, MSC 2x, MMF, tacrolimus	No event	Alive and well
4	M	15	MDS-AML	6/6 CB	ATG, Clo-Bu-Flu	Yes	24	2	3	Pred, CsA	Relapse	Deceased
5	F	9	AML relapse	6/6 CB	Clo-Bu-Flu	Yes	35	2	3	Pred, CsA, MMF	No event	Alive and well
6	M	17	ALL	5/6 UCB	Clo-Bu-Flu	Yes	45	3	3	Pred, CsA, MMF, tacrolimus, sirolimus, monoclonals, MSC, etanercept, surgery	No event	Alive and well
7	F	17	MDS-RAEB-T	10/10 BM	ATG, Bu-Flu	No	41	4	4	Pred, CsA, MSC 3x	No event	Alive
8	F	11	ALL relapse	10/10 sib BM	Bu-Flu	No	24	4	4	Pred, CsA, MMF, MSC 4x	TRM: GVHD	Deceased
9	F	16	Metabolic	4/6 CB	ATG, Bu-Flu	No	39	4	4	Pred, CsA, MMF, monoclonals, MSC 5x	TRM: GVHD	Deceased
10	M	2	PID	6/6 CB	ATG, Bu-Flu	No	49	2	3	Pred, CsA, tacrolimus, MMF	TRM: sepsis	Deceased
11	M	1	PID	5/6 CB	ATG, Bu-Flu	No	89	3	3	Pred, CsA, tacrolimus	No event	Alive

*^*a*^EFS, event free survival; T-ALL, T-cell acute lymphatic leukemia; CB, cord blood; ATG, anti-thymocyte globulin; Bu, busulfan; Flu, fludarabine; Pred, prednisone; CsA, cyclosporin A; PID, primary immune deficiency; RTX, radiotherapy; MMF, mycophenolate mofetil; MSC, mesenchymal stromal cell infusions; TRM, tumor related mortality; MOF, multi-organ failure; MDS-AML, myelodysplastic syndrome-acute myeloid leukemia; Clo, clofarabine; UCB, unrelated cord blood; RAEB-T, refractory anemia with excess blasts in transformation; BM, bone marrow. ^*b*^Based on consensus.*

All patients had received first line treatment with prednisone and continuation of calcineurin inhibitors as treatment of acute (GI-)GVHD. Several patients required more extensive treatment with monoclonal antibodies such as basiliximab (anti-Interleukin (IL)-2 receptor, CD25) or infliximab (anti-TNFα), cell therapy with Mesenchymal Stromal Cells (MSC) or even surgery. Only five patients are currently alive, reflecting the high risk profile of patients with acute GVHD. Two patients died due to relapse, two directly due to GVHD and two due to other—possibly GVHD-related—transplant mortality (sepsis a.o.).

### Broad Detection of Virus in Fecal Samples of Patients With a Previous Viral Diagnosis

We first aimed to determine whether ViroCap was able to confirm the presence of viruses that had been previously diagnosed by real time PCR in the same sample. Six patients (Sample ID 1–6) had a prior, real time PCR established viral diagnosis in the fecal (F) sample tested (Tables 1, 2). In all but one patient (patient 6), the previous detection of adenovirus (ADV) and/or norovirus was confirmed using ViroCap target enrichment and the automated Genome Detective data analysis pipeline. Manual verification of the ViroCap by *de novo* alignment to an ADV reference virus genome using Geneious data analysis software did confirm the presence of ADV in all patients, including patient 6.

Additional pathogens were detected using ViroCap in five of six patients (Table 2A). The additional viruses detected included single cases of human rhinovirus (HRV), ADV and alphatorquevirus and two cases of BK polyomavirus (BKV). These results were confirmed upon subsequent real time PCR testing for BKV in patients 2 and 5 and for HRV in patient 5. ADV could not be confirmed in patient 4. No confirmatory testing was performed for the NGS reported alphatorquevirus detection in patient 6, because a PCR assay for this virus was not available in the laboratory.

**TABLE 2A:** Samples with previous diagnosis.

**Sample no.**	**Previous real time PCR result(s) same sample (Ct value)**	**Total number NGS reads**	**Genome Detective - automated pipeline (reads)**	**Coverage% (depth of coverage)**	**Geneious - manual verification (reads)**	**Real time PCR confirmation (Ct value)**	**Previous real time PCR result(s) other samples**
1F	ADV (Ct 35)	1,261,170	ADV C (9,194) ADV A (430)	99.1 (38.3) 65.1 (3.5)	ADV C (9,424) ADV A (1,701)	NP	ADV pos
2F	ADV (Ct 32)	529,140	ADV C (24) BKV (348)	6.9 (1.7) 99.5 (10.8)	ADV C (201) ADV A (45)	BKV (Ct 26)	BKV pos ADV pos
3F	ADV (Ct 24) Noro (Ct 29)	1,632,432	Adeno-associated virus (34,000) ADV C (32,607) Noro (8,835)	96.3 (1164.9) 99.1 (136.1) 97.9 (167.8)	ADV A (212,743) ADV C (34,136) Noro (7,777)	NP	ADV neg
4F	Noro (Ct 21)	1,570,098	Noro (26,063)	95.3 (492.1)	Noro (7,806) ADV A (15)	ADV neg	Noro pos ADV neg
5F	ADV (Ct 20)	11,635,812	ADV A (8,139,232) BKV (171) HRV-A (39)	99.5 (40,229.4) 96.1 (7.7) 4.3 (1.1)	ADV A (96,108) HRV-C (18)	BKV (Ct 26) HRV (Ct 37)	ADV pos BKV pos (urine)
6F	ADV (Ct 41)	660,224	Alphatorque virus (324)	96.1 (12.8)	ADV A (182)	NP	ADV neg

*^*a*^ADV, adenovirus; BKV, BK polyomavirus; Noro, norovirus; NP, not performed; HRV, human rhinovirus.*

### Viruses Identified in Fecal Samples Without Prior Diagnosis by Real Time PCR

Subsequently, fecal samples of 5 patients which had been tested negative for the presence of ADV, norovirus and rotavirus by real time PCR, direct enzyme immunoassays (EIA) or immune chromatographic testing (ICT), were tested using the ViroCap target enrichment (Sample ID 7–11) (Table 2B). In addition to the aforementioned diagnostic tests, patient 9 had also been tested and found negative by real time PCR for astrovirus, enterovirus and parechovirus. In all but one patient, one or more viruses were detected in the fecal samples. In individual patients we detected single cases of BKV, KI polyomavirus (KI virus), human herpes virus 7 (HHV-7), astrovirus, and alphatorquevirus. HRV was detected in 2 individuals.

**TABLE 2B:** Samples without previous diagnosis.

**Sample no.**	**Previous real time PCR result(s) same sample (Ct value)**	**Total number NGS reads**	**Genome Detective - automated pipeline (reads)**	**Coverage% (depth of coverage)**	**Geneious - manual verification (reads)**	**Real time PCR confirmation (Ct value)**	**Previous real time PCR result(s) other samples**
7F	ND	42,172	BKV (1,808)	4.3 (36.4)	BKV (2,037)	BKV neg	BKV pos
			HHV-7 (6)	NA	HHV-7 (126)		HHV-6 pos
8F	ND	88,962	HRV-C (8)	9.5 (6.7)	HRV-C (631)	HRV (Ct 30)	–
9F	ND	106,936	–	–	–	–	–
10F	ND	603,938	HRV-B (918) Astrovirus VA3 (849) KI virus (301)	48.4 (52.9) 99.9 (25.7) 100 (21.4)	HRV-B (817) Astrovirus VA3 (6055) KI virus (534)	HRV (Ct 18) Astrovirus neg	–
11F	ND	1,856,410	Alphatorque virus (2,055)	22.8 (272.3)	–	–	–

*^*a*^ND, no diagnosis; HHV, human herpes virus; BKV, BK polyomavirus; NA, not available; HRV, human rhinovirus; KI virus, KI polyomavirus.*

The number of reads were generally low and varied between 6 and 6,000. In most cases the read counts were higher upon manual alignment using Geneious software in comparison to the automated Genome Detective pipeline. ViroCap detection of most of the viruses could be confirmed in available real time PCR assays. The HRV detections were confirmed at a Ct value of 18 for patient 10 and Ct 30 for patient 8, despite the low number of NGS reads reported by ViroCap for the HRV of patient 8. The detection of BKV by ViroCap in patient 7 could not be confirmed by real time PCR. However, this patient did have a high viral load of BKV in urine, close to moment of feces collection, as had been observed by routine real time PCR monitoring. No confirmatory real time PCR testing was performed for alphatorquevirus on sample 11F, KI virus on sample 10F and for HHV-7 on sample 7F.

### ViroCap Detects a Recent Astrovirus Variant

**FIGURE 1:**
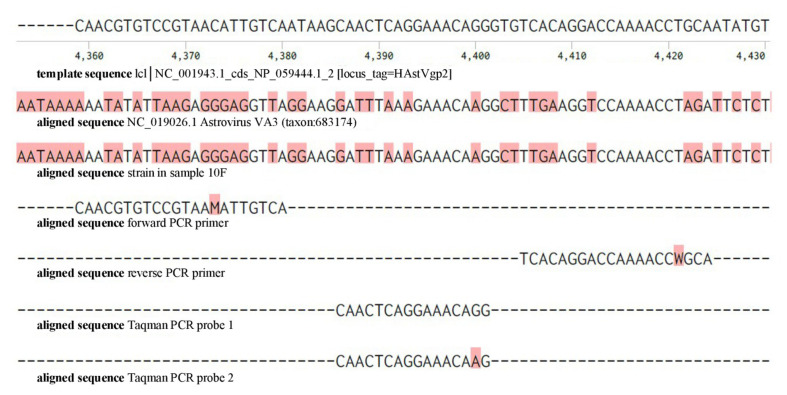
Detailed presentation of the nucleotide sequences of the target region of the astrovirus and real time PCR used for routine application. Top row: template sequence coding for human astrovirus capsid precursor protein (HAsrVgp2), aligned with Astrovirus VA3 sequence and the ViroCap detected astrovirus sequence in sample 10F. Mismatches with the template sequence are indicated in red. Forward PCR primer, reverse PCR primer and Taqman PCR probes applied in the diagnostic astrovirus PCR are indicated in the bottom lines of the figure.

In patient 10F the abundant presence of astrovirus VA3 sequences was reported by Genome Detective. The inherent design of ViroCap enrichment probes containing multiple conserved regions of a virus genome allowed for the detection of this recently described new species of astrovirus VA3 (JX857868.1) (Finkbeiner et al., 2009). The presence of astrovirus could not be confirmed using our diagnostic real time PCR assay. Detailed analysis of the reported NGS sequences revealed that the genome of this specific astrovirus clade VA3 contained extensive mutations in primer regions used in our diagnostic real time PCR assay explaining the failing PCR confirmation (Figure 1).

## Discussion

The role of the intestinal microbiome in the development of GVHD has been a major field of study in the HSCT setting, but reports have mainly focused on the dynamics of bacteria (Shono et al., 2016; Takashima and Hanash, 2017; Shono and van den Brink, 2018; Peled et al., 2020). Besides better known implications of some specific viruses post HSCT, such as ADV (Shields et al., 1985; Flomenberg et al., 1994; Lindemans et al., 2010; Kosulin et al., 2016; Pochon and Voigt, 2018), norovirus (Roddie et al., 2009), and HHV (Sehrawat et al., 2018), to date only one study has explored the gut virome in HSCT using NGS (Legoff et al., 2017). Albeit the promise of unbiased virome mapping, virus discovery with NGS in clinical samples has been hindered by relatively lower sensitivity compared to real time PCR (Sauvage et al., 2016). A study comparing the diagnostic efficiency of NGS vs. gold standard real time PCR in 89 nasopharyngeal swabs reported a sensitivity of 78% and specificity of 80% for NGS (Thorburn et al., 2015). More recently, a NGS sensitivity of 92% compared to real time PCR was found when testing a range of 52 clinical samples, including 8 of fecal origin (Huang et al., 2019).

Target enrichment for sequences of viruses infecting vertebrate organisms, using biotinylated capture probes as a front-end procedure of NGS-based metagenomic sequencing, provides an opportunity for sensitive, broad detection of viruses (Wylie et al., 2015). In two sets of clinical samples (including 1 stool, 7 nasopharyngeal swabs and 1 plasma sample) applying ViroCap resulted in a median fold-increase of the viral reads percentage of 674 and 296, respectively. In the first set, the median breadth of coverage expanded from 2.1 to 83.2% and in the second set from 2.0 to 75.6%. Subsequently, the same authors tested ViroCap in a slightly larger set of 26 clinical samples that were previously submitted to a diagnostic virology lab (including 2 stool samples and in addition whole blood, plasma, cerebrospinal fluid, nasopharyngeal swabs, tracheal aspirates, and skin swabs) and found a consistent increase in the number and percentage of viral reads as well as breadth and depth of viral genome coverage (Wylie et al., 2018). Here we applied ViroCap capture-based enrichment to test virus presence in residual stored clinical stool samples of immunocompromised pediatric patients that had undergone a HSCT in an independent institute and were able to show its advantages even in a small cohort of patients.

ViroCap was capable of detecting all viruses that had previously been detected by pathogen-specific real time PCR assays, proving the robustness and sensitivity of the method. In ADV positive samples the number of NGS reads for ADV was higher at low PCR Ct values and vice versa, but this trend was not statistically significant (data not shown). In some cases we observed differences in the gross number of reads generated with the automated Genome Detective pipeline when comparing with manual alignment in Geneious software. This might be due to differences in the reference sequences used by both programs, an unbalanced representation of sequenced genome fragments or a combination of these factors.

ADV was the most prevalent detected pathogen in our modest patient cohort, in 5 out of 11 patients, which was similar to other reports on HSCT recipients (Yolken et al., 1982; Troussard et al., 1993; Chakrabarti et al., 2000) and immunocompromised patients (Monaco et al., 2016) and non-human primates in general (Handley et al., 2012). Systemic ADV reactivations are notoriously deadly in the pediatric HSCT setting (Lindemans et al., 2010; Kosulin et al., 2016; Pochon and Voigt, 2018), for example the detected ADV C in 3 of our patients has been linked to multiple fatalities (Mynarek et al., 2014). Exemplifying the broad detection potential of ViroCap, several other viruses for which the samples had not been previously tested, were detected. These included HHV-7 (*n* = 1), BKV (*n* = 3), ADV (*n* = 1), HRV (*n* = 3), alphatorquevirus (*n* = 2), KI virus (*n* = 1), and astrovirus (*n* = 1). The implications of some of the aforementioned viral presence, and possible others, is debated and yet to be fully determined. Alphatorquevirus, for instance, is considered to be an apathogenic virus to humans and its DNA has been detected in various clinical samples, including stool, in up to 90% of tested healthy and diseased individuals (Matsubara et al., 2000; Ssemadaali et al., 2016). Nonetheless, a relationship between alphatorquevirus peripheral blood titers and post HSCT complications has been suggested (Gilles et al., 2017). Others, like HHV-7, BKV, KI virus, and HRV, have not yet been associated with GI symptoms or gut GVHD. In general, relatively mild viral infections in healthy individuals can be prolonged or more severe in immunocompromised children. If undetected they may spread among transplanted patients which could potentially lead to a clinical manifestation (Schwartz et al., 2011; Swartling et al., 2015; van der Doef et al., 2016). Interestingly, the mere presence of certain viruses in the gut both before HSCT (van Montfrans et al., 2015) and before or within 1 week after HSCT (Legoff et al., 2017) can be predictive of/predispose for the development of intestinal GVHD. Montfrans analyzed stool samples of 48 pediatric allo-HSCT patients using real time PCR before allo-HSCT and found that the presence of virus (ADV, norovirus, parechovirus or astrovirus combined) predisposed for the development of acute enteric GVHD, but not chronic GVHD (van Montfrans et al., 2015). All viral positive patients remained positive for over 3 months post-HSCT. Similar associations were previously found in our institute between respiratory virus PCR positivity in nasopharyngeal aspirates or bronchoalveolar lavage samples early after transplant and the development of allo-immune lung disease (Versluys et al., 2010). It could however be hypothesized that if investigated with more sensitive and broad techniques such as ViroCap, not only a subgroup, but all HSCT recipients with GI-GVHD are colonized with specific viruses in the gut which may affect HSCT- and GVHD-related outcome.

Legoff and colleagues studied the peri-HSCT gut virome longitudinally using metagenomic NGS on 201 fecal samples collected from 44 HSCT patients (Legoff et al., 2017). The authors demonstrated a progressive increase in the overall proportion of vertebrate viruses in the gut of patients after transplantation, independent from the development of GVHD. However, acute intestinal GVHD patients did experience an increase in persistent DNA viruses, such as anneloviruses and herpesviruses. Additionally, picobirnaviruses (PBVs) were identified in 18 patients, either before or within a week after transplant and its detection pertained predictive of the occurrence of both overall and intestinal GVHD (Legoff et al., 2017). A hypothesis for the described associations is that virus causes mucosal damage, leading to the release of alarmins that activate remaining innate immune cells and increase antigen presentation by host antigen-presenting cells (APC), causing allo-activation and influx of donor T cells (van Montfrans et al., 2015).

Perhaps some clues can be found in mouse studies, where it was postulated that viral presence modulates the occurrence of intestinal bacterial and viral infections in primary immune deficiency models. Latent murine herpes infection protected mice from *Listeria* bacteremia (MacDuff et al., 2015). It was speculated that the chronic infection stimulated the innate immune system such that is compensated for early cytokine response deficiencies in immunodeficiency. More recently, Ingle et al. found that in primary immunodeficient mice astrovirus presence can protect against murine norovirus and rotavirus infections through upregulation of cell-intrinsic IFN-lambda in the intestinal epithelial barrier (Ingle et al., 2019). If these findings are transposed on an allogeneic HSCT setting, it can be postulated that specific viral presence leads to activation of the innate and thereby adaptive immune system, in this setting allo-reactive T cells, and provokes GVHD. In contrast, in recent GVHD mouse model studies, similar innate cytokine signaling pathways activated by viral sequence detection were linked to protection against GVHD. It was shown that activation of the RIG-I/MAVS and cGAS/STING pathways, both innate recognition pathways that induce Interferon type I expression upon sensing of specific viral RNA and DNA sequences, attenuated intestinal GVHD injury (Fischer et al., 2017). Mechanistically, RIG-I activation before HSCT reduced the ability of specific recipient APCs to activate transplanted allogeneic T cells (Fischer et al., 2019). More research is warranted to elucidate the complex correlations between viral presence and the development of GVHD, in which ViroCap could play an important role.

Besides detecting a broader range of viruses than with specific respiratory tract or GI focused PCR panels, ViroCap has the ability to detect viral variants. ViroCap probes cover extensive proportions of the genomes of viral families, species or (sub)types, and as such genetic variants may be well detectable upon capturing the conserved regions of such virus. Nucleotide sequence identity as low as 58% demonstrated to be sufficient for the detection of novel variants (Wylie et al., 2015). In our study, we detected and characterized an astrovirus VA3 that had not been detected by our routine real time PCR assay. The genetic distance of this relatively recently identified astrovirus clade was high and could therefore not be detected in the applied diagnostic qPCR assay. Astrovirus VA3 has been identified rarely in human samples and was specifically reported in the stool samples of a child with diarrhea from India (Finkbeiner et al., 2009). The current identification of this astrovirus clade in our patient cohort of severely immunocompromised symptomatic patients indicates that the potential clinical importance should be considered and further elucidated.

Despite aforementioned benefits, the ViroCap capture-bead technology also has limitations (Wylie et al., 2015; Gaudin and Desnues, 2018). Firstly, the cost of the assay, in particular of the capture probes, is still considerable if only few samples are assayed. Pooling of samples subsequent to the library preparation can help to reduce the assay cost per sample as long as this does not affect the assay sensitivity. In our experiments, we did not observe a reduction in sensitivity upon pooling of up to 10 clinical samples, indicating that the amount of probes per reaction was not a limiting factor (data not shown). With this strategy, the cost per sample can be reduced to 300–400 Euros per sample, not yet comparable to multiple real time PCR. It is expected that the wider application of NGS and of probe capturing strategies will lead to a significant price reduction in the coming years. Furthermore, routine clinical application of ViroCap requires a significant reduction in the assay turnaround time (TAT). Currently, the TAT is in the order of 5 days, mainly caused by the 48–60 hrs required for probe hybridization and 48 hrs of sequencing on the MiSeq system. Commercial reagents reducing hybridization times to less than 4 hrs have recently been introduced and can be considered an important factor for clinical application of the strategy. Finally, ViroCap will not be capable of efficiently enriching viral sequences of variants or sub-species that differ too much from the known species. Nonetheless, since the capture probes cover the full width of vertebrate viruses, the chance of missing a completely new and unidentified viral species of family is limited.

In summary, application of viral target enrichment strategies with limited virus detection bias, such as ViroCap, can lead to the detection of unexpected viruses and viral variants, as demonstrated in the modest number of allo-HSCT patients presented in this manuscript. As such, applying ViroCap to a larger cohort will be a feasible and important next step to elucidate associations of viruses with GI-symptoms and GVHD.

## Data Availability Statement

The sequence data has been uploaded to NCBI - https://www.ncbi.nlm.nih.gov/Traces/study/?acc=PRJNA656436.

## Ethics Statement

Retrospective analysis was performed on long term stored human residual fecal materials, in accordance with the guidelines of the University Medical Center Utrecht on the use of residual diagnostic material for research purposes. The legal guardians of the patients and the patients of legal age themselves provided written informed consent for the use of the participant’s clinical data and diagnostic results.

## Author Contributions

SJ performed the integration of NGS results and clinical information, and prepared the manuscript. WN performed experiments. HL and AR-B provided input to the project design and experiment interpretation. CL and RS initiated and designed the project, supervised the experiments, the interpretation and manuscript preparation. All authors contributed to the article and approved the submitted version.

## Conflict of Interest

The authors declare that the research was conducted in the absence of any commercial or financial relationships that could be construed as a potential conflict of interest.

## Footnotes

1 www.genomedetective.com, Belgium

2 www.geneious.com, United States
